# Supplementary material for: Using Stable Sulfur Isotope to Trace Sulfur Oxidation Pathways during the Winter of 2017–2019 in Tianjin, North China
Source: Int J Environ Res Public Health. 2022 Sep 2;19(17):10966. doi: 10.3390/ijerph191710966 (PMC9518053; doi:10.3390/ijerph191710966)
Supplement: Supplementary file 1 [file ijerph-19-10966-s001.zip › ijerph-1859478-supplementary.pdf]

Supplemental material

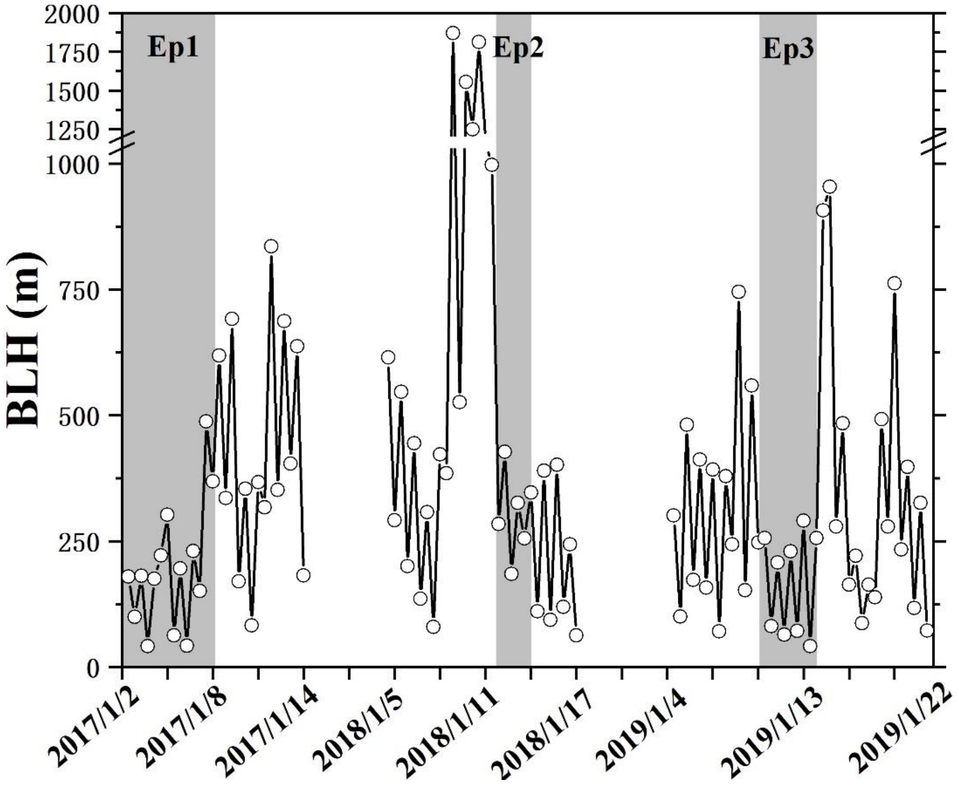

Figure. S1. Time series of BLH during January 2017, 2018 and 2019.

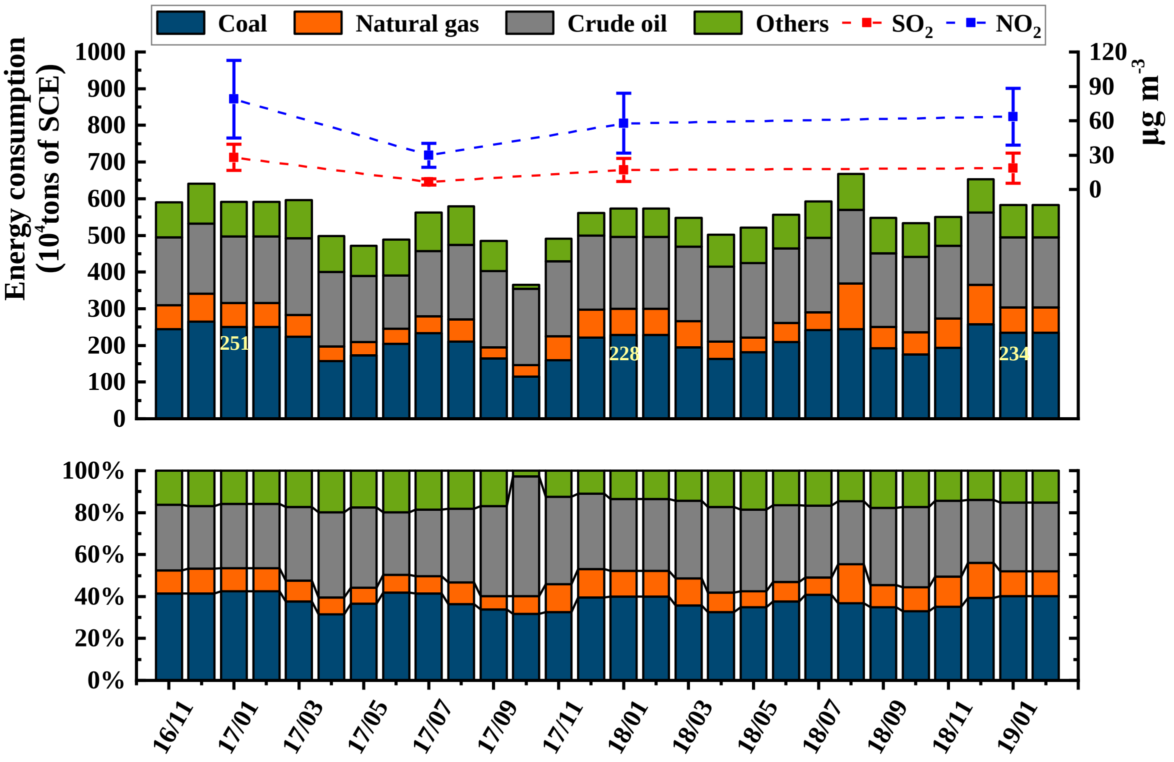

Figure. S2. Consumption of electricity and industrial energy above scale, including coal, natural gas, crude oil, and others (coke, oil product, and thermal) in Tianjin from Jan-2016 to Jan-2019. Data from Tianjin Municipal People's Government. Values for January and February each year are average consumption estimated by the sum of them. The SO_2 and NO_2 data during the sampling period come from the Air Quality Monitoring Station.

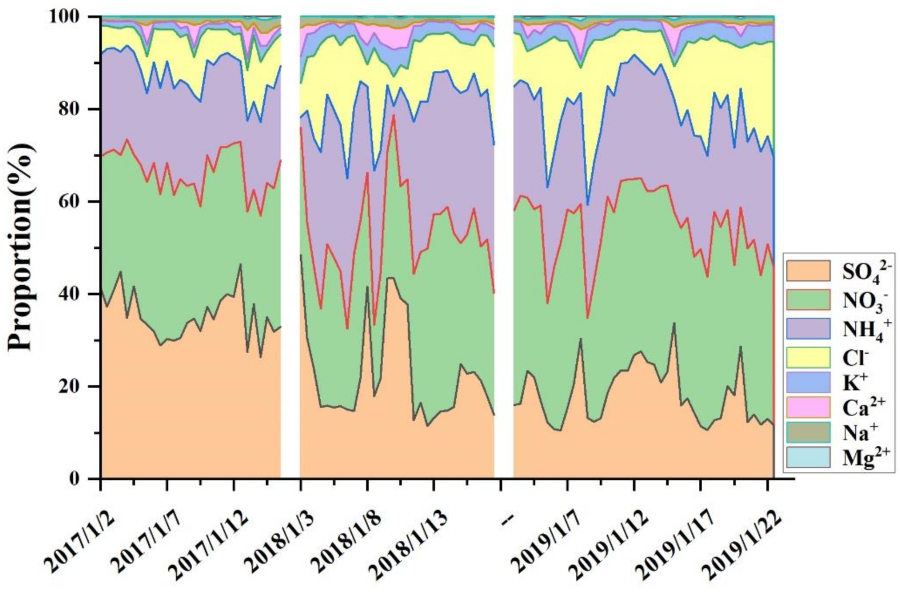

Figure. S3. Proportion of water-soluble ions in $PM_{2.5}$ during sampling period.

Table.S1. Features of sampling phases

Abbreviation	Period	Season	CRP
Jan-2017	1/2~1/15/2017	winter	before
Jan-2018	1/3~1/17/2018	winter	1 year later
Jan-2019	1/3~1/22/2019	winter	2 years later

Table S2. The concentration of $PM_{2.5}$, SO_2, SO_4^{2-} ($\mu g/m^3$), and $\delta^{34}S_{sulfate}$ values of air mass from different clusters.

	Cluster	$PM_{2.5}$	SO_2	SO_4^{2-}	$\delta^{34}S_{sulfate}$
2017/01	1	157.4	29.7	24.9	4.9
	2	215.9	32.5	42.5	5.2
	3	146.5	33.0	19.8	7.4
	4	55.5	12.8	4.1	4.7
	5	73.5	21.5	4.9	6.3
2018/01	1	30.5	12.6	2.7	6.1
	2	77.7	21.8	6.5	5.3
	3	23.7	6.7	1.7	6.1
	4	125.9	26.0	9.9	5.2
2019/01	1	72.2	22.1	5.8	4.7
	2	43.5	14.9	2.8	4.8
	3	114.2	22.9	12.7	3.2
	4	18.8	8.4	1.3	6.1

Table S3. The contribution and uncertainties of different oxidation pathway

	Ep1		Ep2		Ep3	
	f_i (%)	std	f_i (%)	std	f_i (%)	std
OH	25.9	0.2	5.3	0.05	18.0	0.1
H_2O_2/O_3	21.2	0.1	4.2	0.04	17.2	0.1
TMI	24.7	0.1	72.6	0.1	32.3	0.1
NO_2	28.2	0.2	17.9	0.1	32.5	0.2
